# An* In-silico* Approach and Experimental Analysis Combination: Two Strategies for Selecting the third Extracellular Domain (D-EC3) of Human CD133 Marker as a Target for Detection of Cancer Stem Cells

**DOI:** 10.22037/ijpr.2021.115662.15470

**Published:** 2021

**Authors:** Sepideh Ghani, Fatemeh Yarian, Mojgan Bandehpour, Bahram Kazemi

**Affiliations:** a *Department of Medical Biotechnology, School of Advanced Technologies in Medicine Shahid Beheshti University of Medical Sciences, Tehran, Iran. *; b *Department of Medical Biotechnology, School of Advanced Technologies in Medicine, Fasa University of Medical Sciences, Fasa, Iran. *; c *Cellular and Molecular Biology Research Center Shahid Beheshti University of Medical Sciences, Tehran, Iran.*

**Keywords:** In-silico analysis, CD133 (prominin-1), third extracellular domain (D-EC3), Cancer stem cells

## Abstract

The selection of the appropriate fragment of the cell surface receptors as an antigen is significant for the production of antibodies. CD133, as a suitable biomarker candidate in the cancer stem cells (CSCs), is a glycosylated protein. The antibodies used for analyzing it recognize glycosylated epitopes of CD133. Since the glycosylated motifs have a dynamic nature over the lifetime of a protein, they limit the detection of CD133. In this study, to access a specific antibody against the antigenic, accessible, and non-glycosylated fragment of the native CD133, we performed an *in-silico* analysis. Then, we expressed the third domain (D-EC3) (serine641-leucine710) in *E. coli* BL21 (DE3), then the purified recombinant antigen immunized BALB/c mice. Finally, the dignity of an epitope of pure recombinant antigen has been approved by the interactions of antibody and antigen with the use of mice immunized sera via ELISA and flow cytometry experimentation. The results showed that the selected non-glycosylated fragment can compete well with the commercial antibody against the glycosylated epitopes to identify the native cell surface markers. The results can be considered for diagnosis and target therapy development of CD133+ cancer cells.

## Introduction

Emerging evidence has shown that cancer remains one of the major challenges for human health worldwide despite extensive research. Chemotherapy and radiotherapy are the most common treatments against tumorigenesis, but the main challenge is the side effects, recurrence, metastasis, and tumor resistance to these therapies (1). Cancer stem cells (CSCs), a small population of tumor tissue cells, have the unique abilities of self-maintenance and self-renewal (2). Various studies have shown that CSCs are responsible for metastasis and tumor resistance to common therapies (3). Therefore, CSCs identification, isolation, and target therapy could be considered as an efficient therapeutic approach (4).

The cluster of Differentiation 133 (CD133, prominin-1) has been studied to identify CSCs in different types of tumors and is considered one of the prognostic markers of tumor development (5). Also, because of the high expression level of CD133 in the cancer stem cells, it has been proposed as an appropriate candidate for targeting the CSCs (6). CD133, a pentaspan transmembrane glycoprotein, comprises an intracellular C-terminal (IC3) domain, an extracellular N terminal (EC1) domain, 2 glycosylated extracellular (EC2 & EC3) loops, as well as 2 intra-cellular (IC1 & IC2) loops (7, 8). It contains 865 amino acids, and its molecular weight is 120 kDa (9, 10). 

Several methods such as immunohistochemistry and flow cytometry have been used antibodies to identify the CD133-expressing cancer cells (8). On the other hand, the antibodies (AC141, AC133, and 293C3) that are used for the CD133 analysis recognize glycosylated epitopes of the CD133 native protein (11-13). Glycosylated motifs have a dynamic nature over the protein’s lifetime, so analysis based on them yields variable outcomes (11, 14). Also, studies on CD133 have not developed well because of the presence of extracellular glycosylated domains and different splice variants and the lack of antibodies to identify the non-glycosylated regions. Therefore, the aim of this study was to produce improved antibodies against non-glycosylated domains to be used in the diagnosis and treatment of cancers (15, 16).

To select the appropriate fragment of CD133 protein as an antigen for antibody production, it should be noted that produced antibodies must detect the recombinant antigen and the CD133 protein at the cell surface. For this purpose, the EC3 loop of CD133 protein was selected for the study. Due to to the EC3 glycosylation, *in-silico* analysis was used to select the appropriate antigenic, accessible, and non-glycosylated fragment of the EC3 loop for expression in the prokaryotic host cell. The results of this study can be applied in the diagnosis and targeted therapy of cancers.

## Experimental

*Bioinformatics Analysis*

*Protein sequence retrieval and Prediction of Bcell epitopes*

The sequence of the EC3 loop of CD133 (accession no. O43490) was obtained from the UniProt database at (https://www.uniprot.org/) and was used for analysis. The EC3 sequence was investigated with the IEDB analyses resource (http://www.iedb.org): BepiPred linear epitope, flexibility, surface accessibility, and antigenicity prediction have been utilized for detection of the potential B-cell epitopes as well as choosing suitable sequences. Also, the ElliPro server (http://tools .iedb.org/ellipro/) (17) has been used to predict the conformational (discontinuous) B-cell epitopes. Based on these sequence analyses, we selected the domain from EC3 (D-EC3).

*Features evaluation of D-EC3 determinants*

To evaluation of physicochemical parameters of D-EC3 like the molecular weight, theoretical pI, formula, atomic composition, the composition of amino acids, the total numbers of the residue with positive and negative charges, approximated half-life, aliphatic index, grand average of hydropathicity (GRAVY), and instability index the ProtParam tools at (https://web.expasy.org/protparam) (18) was used. 

Also, antigen probability and solubility of D-EC3 were calculated using the VaxiJen (http://www.ddgpharmfac.net/vaxijen/VaxiJen/VaxiJen.html) (19) and Scratch Protein Predictor (http://scratch.proteomics.ics.uci.edu/) respectively (20).

*Prediction of secondary and tertiary structure*

For secondary structures prediction of D-EC3, the SOPMA server was applied. The 3D structure of this fragment was predicted with I-TASSER online server (21). The quality and validity of the model were confirmed by RAMPAGE (22) and ProSA- server (23). 

*Codon Optimization and design of D- EC3 into pET-29 b (+)*

The sequence of the D- EC3 protein has been back-translated to DNA, and then optimization has been performed to be expressed in *E. coli* by gene optimization software (http://www.jcat.de/). Moreover, we computed the Codon Adaptation Index (CAI) and GC content as the approximate indicator of the probable success of expressing the heterologous genes. The D- EC3 gene was designed with the (S)-tag at their N-terminal site and then ordered for synthesis into the expression vector pET-29 b (+) (Novagen, USA).

*Experimental Analysis*

*Expression of the D- EC3*

The resulting plasmid, pET29b/ D-EC3, has been transformed into *E. coli* *BL21 *(DE3) competent-cells by the heat shock. Moreover, colonies have grown on LB culture medium consisting of 30 μg/μL kanamycin (Merck: Germany). The positive mono colonies have been grown over-night in the LB media consisting of 30 μg/μL kanamycin and incubated at 37 °C (150 rpm, 16 h). The culture was diluted in a fresh medium with 15 μg/μL kanamycin for selection and cultured for 2 h at 37 °C till absorbance reached 0.6 at 600 nm. IPTG (Isopropyl-beta-D-thiogalacto-pyranoside) (Merck: Germany) has been poured into the culture at the resulting concentration of 1 mM as the inducer and samples have been gathered 0, 2, 4, and 6 h following the induction. These cells have been harvested, and centrifugation has been done at (8,000 rpm, 5 min), given treatment with lysis buffer (50 mM Tris base, 0.1% Triton X-100, 10% glycerol) manufactured by Merck Co., Germany, sonication of the cell lysate has been performed on ice, precipitated with acetone (overnight, -20 °C), and centrifugation has been done at 6,000 rpm: 5 min; 4 °C). This lysate has been analyzed by electrophoresis on a 15% SDS-PAGE; that is, sodium dodecyl sulfate-polyacrylamide gel electrophoresis) Moreover, protein bands have been stained with the coomassie brilliant blue (24). 

*Western blot analysis of recombinant protein*

According to the research design, the protein was transported into a nitrocellulose membrane from SDS–PAGE gel (Wathman, UK). The membrane was blocked with TBS buffer (Tris-buffered Saline) consisting of 3% Bovine Serum Albumin (BSA) (Sigma; USA) and incubated with 1:10000 dilution of the alkaline phosphatase (ALP) conjugated anti-S-tag monoclonal antibody provided from Abcam; the UK, at the room temperature for two hours. Finally, the specific band was revealed by NBT/BCIP substrate solution (Roche, Germany)(25).

*Affinity Chromatography to purify the recombinant D-EC3 protein*

 In this stage, the recombinant D-EC3 protein has been purified using a column with S-protein Agarose by the manufacturer’s instructions (Novagen, USA) and then dialyzed with PBS (pH 7.4) for 8 h at 4 °C. Finally, the protein was concentrated by freeze-drying. Western blotting and SDS-PAGE have been used to analyze the protein purity with an antiS-tag antibody.

*Immunization of the BALB/c mice with the recombinant D-EC3* *protein*

In compliance with all ethical principles of research on laboratory animals following the animal ethics guidelines of the Committee and code of ethics (IR.SBMU.RETECH.REC.1396.1300), three female 4-week-old BALB/c mice were obtained from the Pasteur Institute (Tehran, Iran). They were injected intraperitoneally with a 1:1(v/v) mixture of 50 (μg/ mL) recombinant protein with S-tag peptide and alum adjuvant (Sigma, USA). The second and third infusions were performed 12 and 14 days after the first injection with the same amount of antigen and alum adjuvant, respectively. A mouse as control was injected with the same proportion 1:1(v/v) of phosphate-buffered saline (PBS) and adjuvant. Then, the mice’s sera were analyzed by ELISA and Flow cytometry.

*ELISA experiment*

ELISA was used to analyze the interaction of antigen with the antibody. Then, the ELISA plate was coated with 100 μL of the recombinant D-EC3 protein conjugated with 2 μg/mL S-Tag peptide. In the next step, over-night incubation was done at the room temperature, and upon the washing with PBST (PBS with 0.05% Tween 20), we blocked this plate with 100 μl/ well of 1% BSA, and incubation has been done at a temperature of 37 °C for two hours. When washed in triplicate with PBST, we poured 100 μL of the mouse serum (diluted 1/100 and 1/500 in PBS) into all wells and incubation was performed at room temperature for two hours. Afterwards, 100 μL of goat antimouse Horseradish peroxidase (HRP) conjugate, as the secondary antibody, 1:10000 in the PBS was poured into all wells and then incubated at a temperature of 37 °C for two hours. In this step, it has been rinsed in triplicate. Ultimately, 100 μL of tetramethylbenzidine (TMB) substrate provided from Sigma in Germany has been poured into all wells. Incubation has been done at the temperature of 37 °C for five minutes in the dark and then this reaction has been ceased via the addition of 50 μL of the stopping buffer (H_2_SO_4_ 2 N) to each of the wells. Next, we measured absorbance at 450 nm using an ELISA reader. The sera of mice injected with PBS and BSA (2 μg/mL) were considered controls, and testing has been accomplished for each sample three times(26).

*In-vivo binding examination*

We used density gradient centrifugation to isolate the peripheral blood mononuclear cells (PBMCs) with a Ficoll-Paque solution according to the research design. At first, the PBMCs were collected (2 mL) in a tube containing Ficoll solution (2 mL) and centrifuged at 2000 rpm at 25 °C for 10 min in a swinging-bucket rotor that had no brake. Then, sterile PBS was applied for washing the harvested PBMCs, and the cells were suspended in the ice-cold PBS consisting of 4% FBS (2.5 × 10^5^ cells in 100 µL). After that, the diluted immunized BALB/c mice serum (1/50) and the diluted normal BALB/c mice serum (1/50) were transported into the assay tubes consisting of 2.5 × 10^5^ cells as test and negative control primary antibodies, respectively. Incubation of the tubes has been done at a temperature of 4 °C for one hour, and PBS with 4% FBS has been used to wash the cells two times, and centrifugation has been performed at 1000 rpm for five minutes. Then, the cell pellets have been re-suspended in PBS, 4% FBS consisting of the FITC-conjugated antimouse IgG (1:1000) (Sigma; Germany) as a secondary antibody for test and negative control. In the next stage, we incubated it at a temperature of 4 °C in the dark for thirty minutes. Also, the anti-CD133 PE conjugate (1:1000) from Sigma in Germany) used for positive control. A FACS Calibur flow cytometer (BD Biosciences) was employed to analyze the staining cells.

## Results

*In-silico analyses of the D-EC3 loop from the CD133 protein*

**Figure 1:**
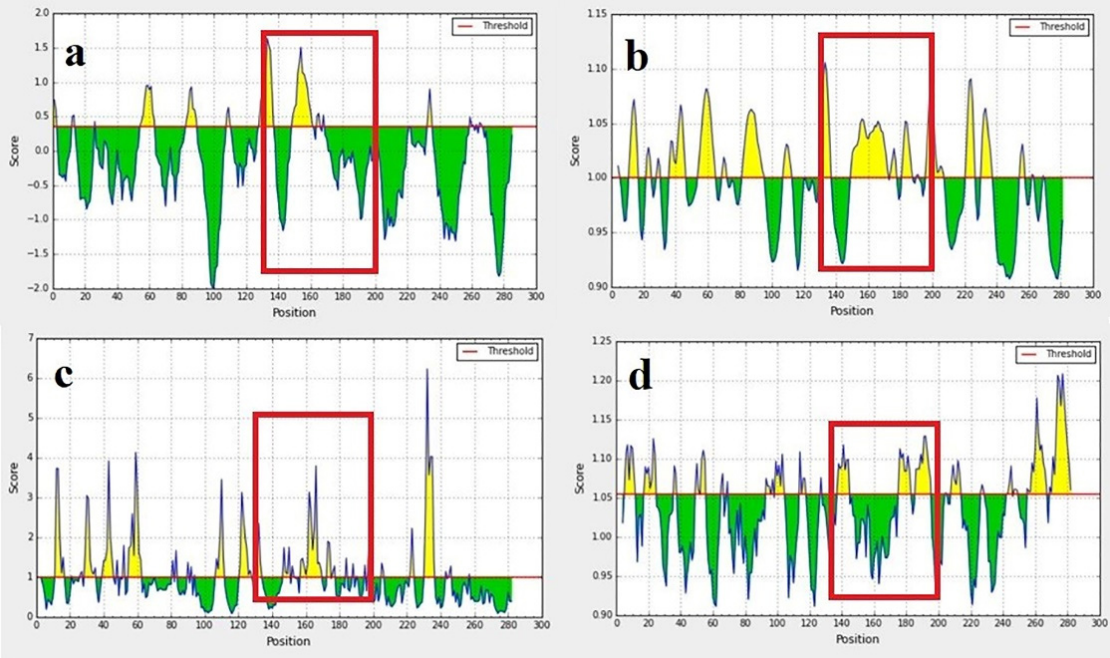
B-cell epitope prediction: BepiPred linear epitope (a) flexibility, (b) surface accessibility, (c) and antigenicity prediction, (d) of EC3. The threshold is represented by the horizontal line. The area under the threshold refers to the undesirable sections of the respective features. The area over the threshold shows desirable areas with the greater-scored residues. Selected regions showed with the red box

**Figure 2:**
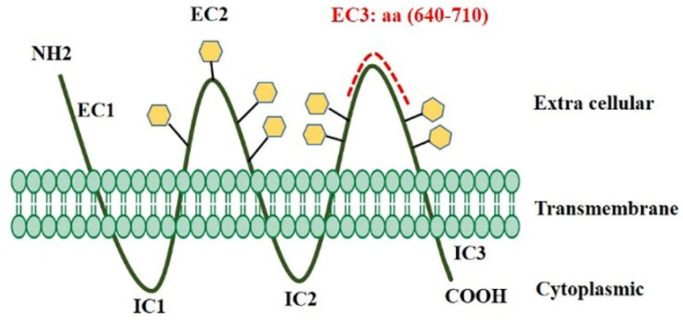
The topological map of CD133 protein used for mAb production. D-EC3 of CD133 protein consisting of amino acid residues 640–710 (red dotted line) was generated

According to information obtained from the UniProt database, EC3 has 284 amino acids (508-792), and this loop contains four glycosylation sites on amino acids 548, 580, 729, and 730. Figure 1 depicts the results of B-cell linear epitope prediction of EC3. The score threshold value is set by default to 1 for flexibility and surface accessibility, 1.05 for antigenicity, and 0.35 for BepiPred linear epitope. In addition, Table 1 reports results of discontinuous B-cell epitopes prediction with the ElliPro server that the minimum score and maximum distance (Angstrom) for epitope identification were defined equal to 0.5 and 6, respectively. Using in-silico analyses and according to the glycosylation pattern of the EC3, the D-EC3 sequence contains 70 amino acids (640-710) with appropriate accessibility and antigenicity was selected (Figure 2). These results showed the antigenicity and the recombinant protein ability to stimulate the mice’s immune system and antibody production.

*Analyzing the D-EC3 protein features*

Total outputs of the chemical and physical properties of D-EC3 are shown in Table 2. The designed domain with a molecular weight 7.7 kDa, was a basic and soluble protein. These properties were significant in prokaryotic expression, whereas its expected half-life was predicted >10 h. The D-EC3 antigenicity with a threshold 0.5 has been anticipated as 0.5396% by Vaxign software. Thus, we may consider the D-EC3 protein probably is an antigenic protein. 

**Figure 3:**
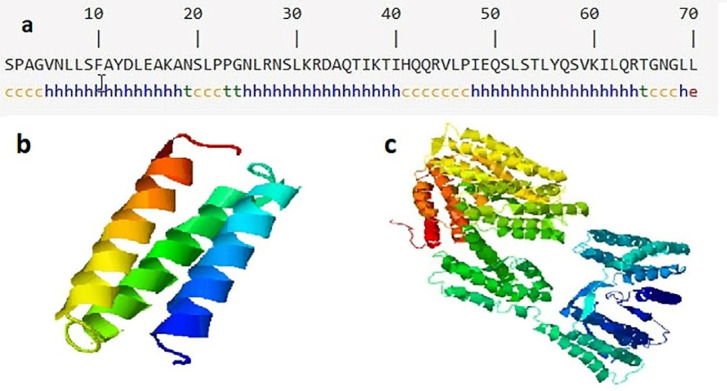
Secondary and tertiary structures of EC3 domain and CD133. (a) The predicted secondary structure of D-EC3 with the use of SOPMA software. H: Alpha helix, E: Extended strand, T: Beta-turn and C: Random coil, (b) The alpha chains of D-EC3 protein. (c) Native CD133 protein by I-TASSER server

The results of secondary structure prediction showed that D-EC3 contained 68.57% alpha-helix (Hh), 1.43% extended strand (Ee), and 5.71% beta strands (Tt), and 24.29% random coil (Cc) (Figure 3). As seen in Figure 3, the natural structure of the CD133 is an alpha-rich protein, and its D-EC3 domain contains three alpha chains. 

We used I-TASSER online web server for the prediction of the 3D structure of the selected sequence (Figure 3). The appropriate model regarding the C-score was chosen (confidence score) to evaluate the anticipated model quality. C-score has been regarded in the scope between -5 and 2 (notably the greater score would be more acceptable) that the D-EC3 and native CD133 protein had a proper quality structure with -1.7 and -0.72 score respectively (16). 

**Figure 4:**
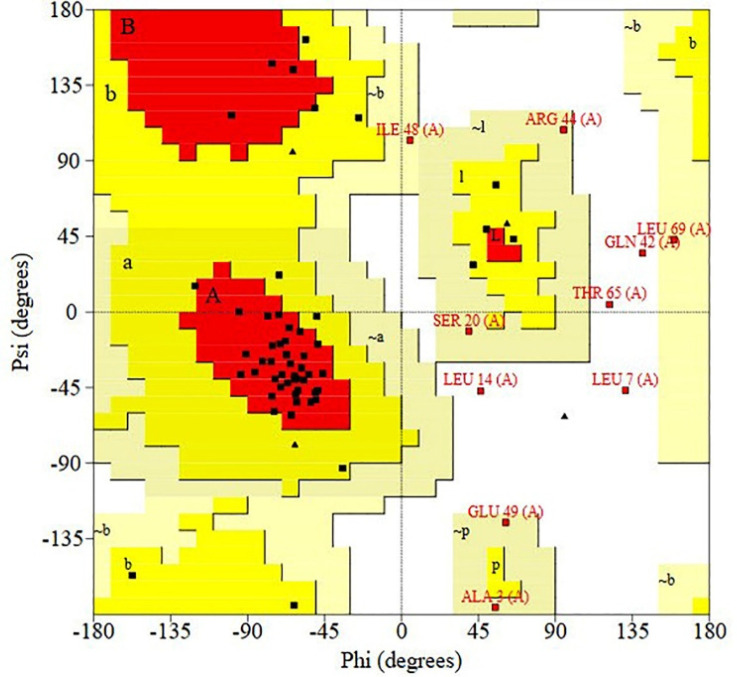
Validating the recombinant D-EC3 with the Ramachandran plot by PROCHECK server. As seen, for D-EC3, 39 residues (65%) have been situated at the desired area, 17 residues (28.3%) in the permissible area, and four residues (6.7%) in the outlier area. 93.3% of the protein residues were in reasonable areas

RAMPAGE server was used to calculate the Ramachandran plot. According to the outputs, 93.3% of the residues for D-EC3 protein existed in reasonable areas (Figure 4). 

**Figure 5:**
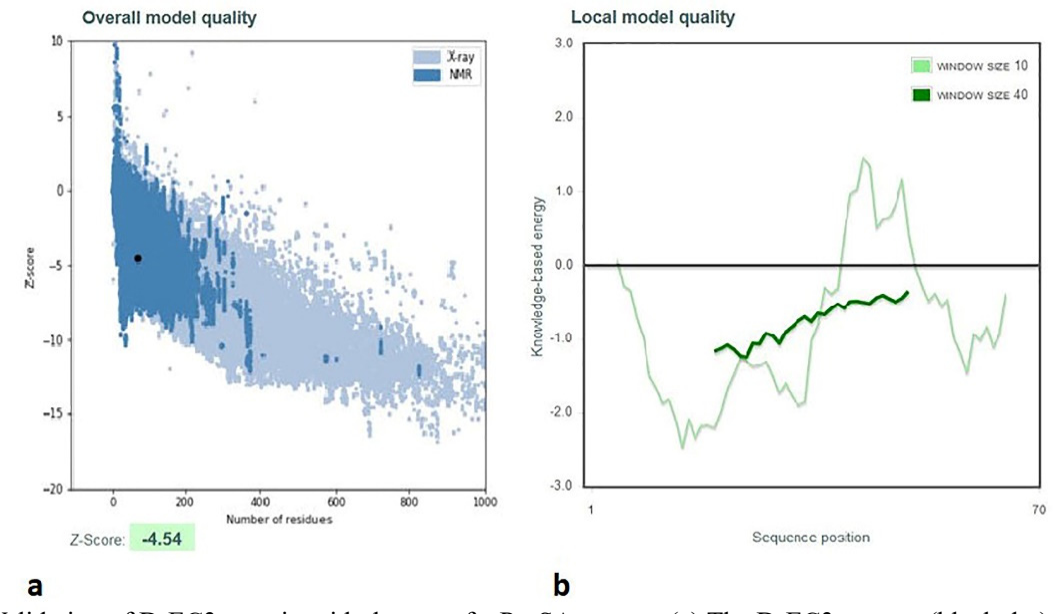
Validation of D-EC3 protein with the use of a ProSA- server. (a) The D-EC3 z-score (black dot) was within the range of scores (– 4.54), usually observed for the native protein with a similar size. z-Score plot consists of the z-score of each experimental protein chain in PDB that has been specified via NMR spectroscopy, which is shown in dark blue, and X-ray crystallography, which is shown in light blue. Moreover, this plot represents the outputs with a z-score ≤ 10. This protein z-score is represented in the large black dot. (b) Energy plots of the D-EC3 model achieved via the ProSA server. Hence, each residue has negative values for the refined model

Additionally, the Z-score of D-EC3 protein (−4.54) demonstrated the anticipated structure’s reliability with the high qualities (Figure 5a). 

According to Wiederstein and Sippl, “the energy plot represented the quality of local model via drawing energies as a function of the amino acid sequence as well as positive-values corresponded to erroneous or problematic parts of the inputs’ structure” (Wiederstein & Sippl, 2007). According to Figure 5b, the energy plot of D-EC3 showed highly negative energy values.

According to the performed analyses, the possible high level of protein expression related to CAI value and a CAI of 1.0 have been considered the ideal value to express in the intended organism of expression. Also, GC’s content has been regarded as one of the measurement values for translational and transcriptional efficiencies wherein the ranges of 30–70% are the desired value (27). Following the optimization process in the present study, the values equaled 1.0 and 53.33% for D-EC3.

*Expression and purification of the recombinant D- EC3 protein*

**Figure 6:**
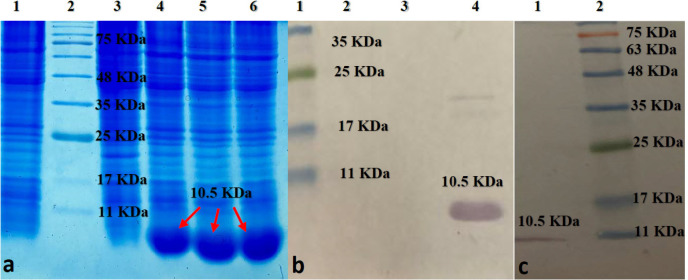
(a) SDS-PAGE analysis of D-EC3 protein expression at different times: Lane1: BL 21; Lane 2:marker ; Lane 3: 0 time; Lane 4:2h ; Lane 5: 4h; Lane 6: 6h after induction. (b) Western blot analysis of D-EC3 protein: Lane 1: marker; Lane 2: BL 21; Lane 3: 0 time; Lane 4, 6h after induction. (c) Western blot analysis of the D-EC3 following the purification: Lane 1: purified protein; Lane 2: marker

Optimized expression of the D-EC3 protein was obtained after six hours after induction at 37 °C (Figure 6a). The western blotting analyses demonstrated the particular bands at a dimension of <11 kDa for D- EC3 that confirmed its expression (Figure 6b). Purification of the D-EC3 protein was carried out using an S-protein agarose column. The expected protein band has been observed in the eluted fraction and approved with the western blot procedure (Figure 6c).

*ELISA experiment*

**Figure 7:**
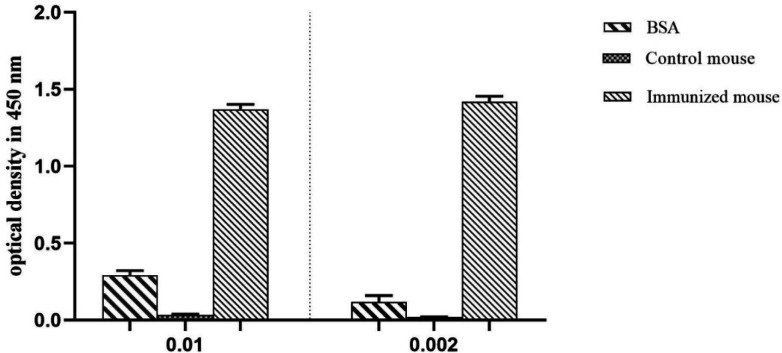
ELISA analysis of mice immune response. Immunized mouse (injected with D-EC3 protein) and control mouse (injected with PBS) sera (1: 100 and 1:500 dilution). ELISA was performed using 2 μg/mL of the purified D-EC3 protein and 2 μg /mL BSA

ELISA was utilized to determine the interaction between antigen and antibody, and a considerable positive signal has been observed in the interaction of the D-EC3 protein with immunized mouse serum compared to BSA and control mouse serum (Figure 7).

*In-vivo binding examination*

**Figure 8:**
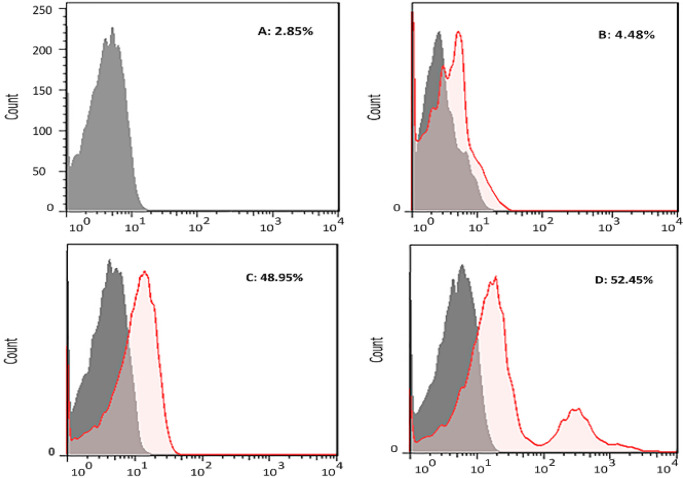
The analysis of the binding of antibody to antigen by flow cytometry. Human PBMCs were treated by (A) Unstained, (B) serum of mouse injected with PBS (1/50 dilution), (C) serum of mouse immunized with recombinant D-EC3 (1/50 dilution) and (D) 2 µg of commercial antiCD133 monoclonal antibodies

The capability of the produced mouse antibodies against D-EC3 recombinant protein in approximately binding to the native human CD133 antigen on the PBMC surface was determined by flow cytometry. Approximately 52.45%, 48.95%, and 4.48% in PBMCs population were stained by anti-CD133 specific mAb (positive control), the serum of mouse immunized with recombinant D-EC3 and the serum of mouse injected with PBS respectively (Figure 8).

**Table 1:** The prediction of conformational B-cell epitope with the ElliPro server

**NO.**	**Residues**	**Number of residues**	**Score**
1	**A:S186, A:T187, A:Q190, A:K193, A:I194, A:Q196, A:R197, A:T198, A:G199, A:N200, A:G201, A:L202,** A:E204, A:R205, A:V206, A:T207, A:R208, A:I209, A:L210, A:A211, A:S212, A:L213, A:D214, A:F215, A:A216, A:Q217, A:N218, A:F219, A:I220, A:T221, A:N222, A:N223, A:T224, A:S225, A:S226, A:V227, A:I228, A:I229, A:E230, A:E231, A:T232, A:K233, A:K234, A:Y235, A:G236, A:R237, A:T238, A:Y242, A:F275, A:L276, A:C277, A:S278, A:Y279, A:I280, A:I281, A:D282, A:P283, A:L284, A:N285	59	0.753
2	A:I100, **A:E148, A:A149, A:K150, A:A151, A:N152, A:S153, A:L154, A:P155, A:P156, A:G157, A:N158, A:L159, A:R160, A:N161, A:S162, A:R165, A:D166, A:Q168, A:T169, A:T172, A:Q175, A:Q176**	23	0.746
4	**A:L179, A:P180, A:Q183**	3	0.635

^*^ Amino acids located in the selected regions are indicated in Bold font.

**Table 2:** Physicochemical parameters of D-EC3 by ProtParam tool

**Physicochemical parameters**	**Loop 3 of CD133**
Molecular weight	7687.83 Da
Numbers of amino acid	70
Theoretical pI	9.99
Formula	C_340_H_565_N_99_O_103_
Total numbers of the residues with positive charge	8
Total numbers of residues with negative charge	4
Expected half-life (*Escherichia coli, in-vivo*)	>10 h
Expected half-life (*yeast in-vivo*)	>20 h
Half-life (*mammalian reticulocytes, in-vitro*)	1.9 h
Instability index	63.36
Aliphatic index	108.71
GRAVY	-0.290

## Discussion

CD133 (prominin-1) is a glycosylated protein over the surfaces of the normal and cancer stem cells that contribute to the proliferation, self-renewal, differentiation, and tumor formation (28). Due to the application of antibodies in immunohistochemistry, immunocytochemistry, and flow cytometry tests used to diagnose cancer cells, the production of specific antibodies is of particular importance (29-31). We preferred to produce recombinant protein as target antigen. The production process in eukaryotic host systems is complex and often protein yield is low, but expressing the recombinant proteins in E. coli cells has been considered easy, cost-effective and highly efficient (32). Glycosylation has been proposed as one of the main parameters for the activity of recombinant proteins. On the other hand, post-transcriptional modifications do not occur in *E. coli* (33). 

CSCs may alter the CD133 glycosylation pattern after differentiation, and if antibodies only recognize the glycosylated epitopes, some CD133 positive cells may not be identified (34). In this study, we used bioinformatics tools to select the non-glycosylated fragment of EC3 as an antigen then we successfully expressed it (a 10.5 kDa-recombinant polypeptide) in the expression system of *E. coli* prokaryotic. 

By binding of immunized mouse serum to the D-EC3 in ELISA, we could approve the integrity of the antigenic epitope in the *E. coli*-derived recombinant protein. According to studies, because the secondary and 3D structures in shorter fragments are more similar to native proteins, shorter fragments could better identify the target proteins (35, 36). Therefore, we selected a short fragment of the EC3 (amino acids serine 641- leucine 710) sequence based on *in-silico* design. There are several papers in the literature which verify our results. Some of the most important ones are listed in the following. Bidlingmaier et al. used the AC133 and AC141 monoclonal antibodies that detect glycosylated epitopes of CD133 protein. Data showed these mAbs could be reported false negative and positive results. Because the glycosylation statusmaybe the criterion for identification instead of the expression of the CD133 protein (11). Also, Kemper et al. used an AC133 antibody for the identification a glycosylated epitope on CD133. Results showed this epitope might be masked because of glycosylation and changes in protein folding. Therefore, using of this antibody should be interpreted with caution(12). Wang *et al.* examined EC2 (amino acids isoleucine171- isoleucine 420) and EC3 (amino acids phenylalanine 507- valine 716) that antibodies produced against EC3 were able to bind the full-length glycosylated CD133 over the tumor cells’ surface (37). EC2 appears that is covered by EC3 and EC1 located on either side of it and is less accessible than EC3. 

Bioinformatics with precise and close-to-reality anticipation at the genes, mRNA, and protein levels; can empower us to improve recombinant protein features, the rate of expression, secondary and tertiary structures, antigenicity, and final solubility (38). Concerning diverse parameters like the linear and conformational B-cell epitopes, a suitable fragment of the target protein can be selected as an antigen. Then it can be confirmed by examining the physicochemical properties, similarities of the secondary and tertiary structures of the selected fragment with native protein.

The ultimate approval of the CD133 antigenic integrity has been shown in the flow cytometric analyses of the CD133-cells fluorescent staining in the PBMC cell samples. This new mouse anti-CD133 D-EC3 pAb stained CD133 cell-population and CD133 mAb (48.95% for mouse anti-CD133 pAb as compared to 52.45% for anti-CD133 FITC mAb). 

## Conclusion

Since both ELISA and Flow cytometry tests verify our designed D-EC3 structure, this structure is a suitable antigenic domain to generate the anti-CD133 monoclonal and poly-clonal antibodies. Produced antibody in mice serum is applicable for diagnosis and cancer target therapies.

## Conflicts of interest

The authors declare that they have no conflict of interest.
